# Microbiome-Derived Indole-3-Lactic Acid Attenuates *Cutibacterium Acnes*-Induced Inflammation via the Aryl Hydrocarbon Receptor Pathway

**DOI:** 10.3390/ijms27031131

**Published:** 2026-01-23

**Authors:** Sang Gyu Lee, Nam Hao Chau, Seoyoon Ham, Yujin Baek, Ngoc Ha Nguyen, Seon Hwa Kim, Young In Lee

**Affiliations:** 1 Department of Dermatology, Cutaneous Biology Research Institute, Yonsei University College of Medicine, Seoul 03722, Republic of Korea; dltkdrb5658@yuhs.ac (S.G.L.); hsy7852@yuhs.ac (S.H.); uj200076@yuhs.ac (Y.B.); ngocha7996@yuhs.ac (N.H.N.); 2 Department of Dermatology, University of Medicine and Pharmacy at Ho Chi Minh City, Ho Chi Minh City 17000, Vietnam; chauhaonamump@gmail.com; 3 Bioinformatics Collaboration Unit (BiCU), Yonsei Biomedical Research Institute, Yonsei University College of Medicine, Seoul 03722, Republic of Korea; stellaaa@yuhs.ac; 4 Scar Laser and Plastic Surgery Center, Yonsei Cancer Hospital, Seoul 03722, Republic of Korea

**Keywords:** acne vulgaris, *Cutibacterium acnes*, aryl hydrocarbon, indoles, keratinocytes

## Abstract

Acne vulgaris is a chronic inflammatory dermatosis where conventional therapies often face limitations in efficacy and safety, necessitating the development of microbiome-targeted interventions. This study investigated the immunomodulatory potential of microbiome-derived tryptophan metabolites as a novel therapeutic strategy for *Cutibacterium acnes* (*C. acnes*)-induced inflammation, focusing on the aryl hydrocarbon receptor (AHR) pathway. We evaluated indole-3-lactic acid (ILA), indole-3-acrylic acid (IAA), and indole-3-propionic acid (IPA) in comparison to tapinarof, utilizing *C. acnes*-stimulated human epidermal keratinocytes and a *C. acnes*-induced acne mouse model. In vitro, ILA and IPA significantly suppressed *C. acnes*-driven inflammatory mediators, including Tumor Necrosis Factor-alpha (TNF-α), Interleukin (IL)-1β, and Cyclooxygenase-2 (COX2), whereas IAA demonstrated limited efficacy. In vivo, ILA treatment exhibited superior therapeutic activity, markedly reducing inflammatory cell infiltration, epidermal hyperplasia, and IL-1β expression. Transcriptomic analysis confirmed that ILA attenuates inflammatory signaling (e.g., IL-17 and TNF pathways) while upregulating AHR-responsive genes such as *Cytochrome* (*CYP*) *1A1* and *CYP1B1*. Collectively, these findings establish ILA as a potent postbiotic that mitigates cutaneous inflammation through selective activation of the AHR. Future studies should prioritize the clinical translation of ILA-based topical formulations, with rigorous evaluation of their efficacy and safety in well-designed human trials, to support their development as a non-antibiotic therapeutic alternative for acne management.

## 1. Introduction

Acne vulgaris is a common chronic inflammatory disease of the pilosebaceous units (hair follicles and sebaceous glands), typically affecting the face, chest, and back in adolescents and young adults. Approximately 85% of individuals between 12 and 24 years of age experience the condition [1]. Clinically, acne is characterized by multiple lesion types, including non-inflammatory comedones (blackheads and whiteheads) and inflammatory papules, pustules, nodules, or cysts that can lead to scarring. Its pathogenesis is multifactorial, involving follicular hyperkeratinization, excess sebum production, colonization by *Cutibacterium acnes* (*C. acnes*), and an aberrant inflammatory response at all stages of lesion development [2].

Standard treatments for acne include topical retinoids, benzoyl peroxide, antibiotics, hormonal therapies, and oral isotretinoin [3]. While often effective, these therapies have notable limitations, including skin irritation, antibiotic resistance, and serious systemic side effects, and their roles are limited in addressing the underlying dysregulation of the skin microbiome [4]. Such limitations have prompted a shift toward alternative therapeutic strategies. In particular, there is a growing interest in the market on microbiome-targeted therapies for acne. Emerging approaches, including natural bioactive compounds, probiotic supplementation, or other microbiome-based interventions, aim to restore a healthy microbial balance and modulate cutaneous inflammation, and early studies indicate they can improve acne outcomes by reducing dysbiosis and inflammatory signaling [5,6,7,8,9]. For instance, essential oils derived from botanical sources such as tea tree, oregano, thyme, and rosemary have demonstrated the ability to suppress *C. acnes*, modulate pro-inflammatory cytokine signaling, and reduce oxidative stress in preclinical and early clinical studies [10]. This paradigm shift reflects a broader recognition of the “gut–skin” and “skin microbiome–skin immunity” axes as promising targets in acne management.

One mechanistic link between the microbiome and skin inflammation in acne is the aryl hydrocarbon receptor (AHR) signaling pathway. Cutaneous AHR activity plays a complex role in immune homeostasis, and dysregulated AHR signaling has been implicated in chronic inflammatory skin diseases such as atopic dermatitis, psoriasis, acne, and hidradenitis suppurativa [11,12]. Notably, certain tryptophan (Trp) metabolites produced by commensal bacteria can bind to AHR in skin-resident cells and exert anti-inflammatory effects. In acne vulgaris, gut microbiome-derived Trp metabolites can regulate skin immune cells to alleviate skin and modulate sebum production via the AHR pathway [13]. Conversely, in hidradenitis suppurativa (HS), skin lesions exhibit an imbalance in Trp catabolism, leading to defective AHR activation, which contributes to uncontrolled inflammation in the skin [14]. These findings highlight the importance of the Trp–AHR metabolic axis in regulating cutaneous inflammation.

Despite increasing recognition of the microbiome–AHR axis in cutaneous immune regulation, the specific roles and comparative anti-inflammatory capacities of individual microbiome-derived Trp metabolites in acne vulgaris remain insufficiently defined. The aim of the present study was not only to evaluate the anti-inflammatory effects of representative Trp-derived indoles in experimental acne models, but also to identify a functionally dominant metabolite with translational relevance. By systematically comparing indole-3-lactic acid (ILA), indole-3-propionic acid (IPA), and indole-3-acrylic acid (IAA) against a clinically validated AHR agonist, and integrating in vitro, in vivo, and transcriptomic analyses, this study provides mechanistic evidence that Trp-derived indoles can uniquely reprogram *C. acnes*-induced inflammatory responses through activation of AHR-associated homeostatic pathways. This work introduces Trp-derived indoles as a previously underexplored group of postbiotic small molecules with selective immunomodulatory activity in acne, thereby advancing current understanding of microbiome-derived metabolites as precision, non-antibiotic therapeutic candidates for inflammatory skin disease.

## 2. Results

### 2.1. In Vitro Cytotoxicity and Anti-Inflammatory Screening of Metabolites

**Figure 1:**
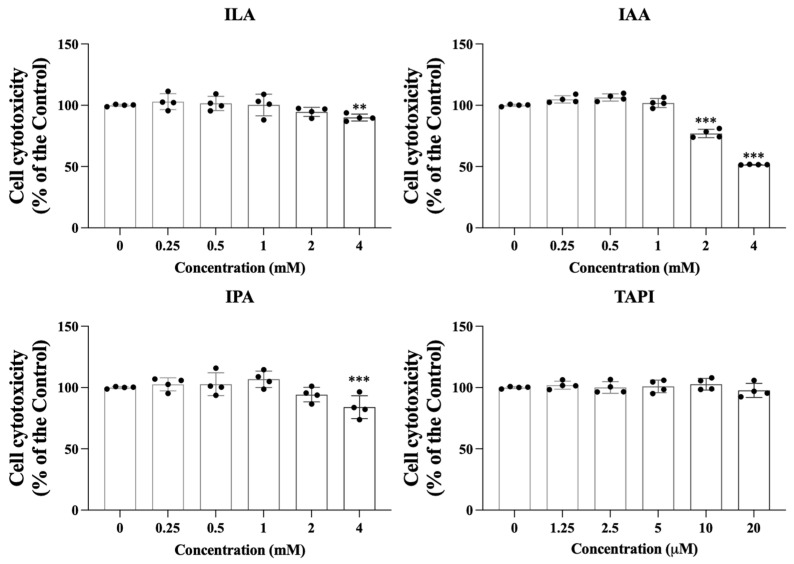
Keratinocytes were exposed to increasing concentrations of ILA, IAA, and IPA (0–4 mM) or tapinarof (0–20 μM) to evaluate the cytotoxic effects of each candidate molecule. Cell viability was quantified relative to untreated controls. Data are presented as mean ± SD. Each dot represents an individual value. ** *p* < 0.01, *** *p* < 0.005, independent samples *t*-test vs. control group, ILA, indole-3-lactic acid; IAA, indole-3-acrylic acid; IPA, indole-3-propionic acid; TAPI, tapinarof; SD, standard deviation.

The cytotoxicity of the candidate metabolites—Indole-3-lactic acid (ILA), Indole-3-acrylic acid (IAA), Indole-3-propionic acid (IPA), and tapinarof (TAPI)—was first evaluated in keratinocytes (KCs) using a CCK-8 assay. As shown in Figure 1, all metabolites maintained >90% cell viability at lower concentrations, indicating minimal cytotoxicity. Viability began to decrease at 4 mM for ILA and IPA and at 2 mM for IAA. Based on these findings, 4 mM ILA and IPA, 2 mM IAA, and 20 μM TAPI were selected for subsequent experiments.

**Figure 2:**
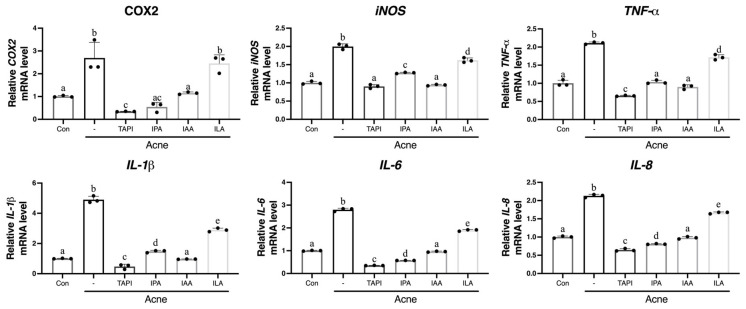
KCs were stimulated with *C. acnes* (Acne group) or co-treated with TAPI, IPA, ILA, or IAA to evaluate their anti-inflammatory activity. *C. acnes* markedly increased the mRNA expression of IL1β, IL6, IL8, TNFα, iNOS, and COX2 compared to the control group. Treatment with TAPI, IPA, and ILA significantly reduced the expression of these inflammatory mediators relative to the Acne group, whereas IAA exerted only a partial or weak suppressive effect. Data are presented as mean ± SD. Different lowercase letters (a, b, c, etc.) indicate statistically significant differences between groups (*p* < 0.05) as determined by one-way ANOVA followed by Tukey’s honestly significant difference (HSD) post hoc test. CON, control; -, Acne group; TAPI, tapinarof; ILA, indole-3-lactic acid; IAA, indole-3-acrylic acid; IPA, indole-3-propionic acid; KC, keratinocyte; *C. acnes*, *Cutibacterium acnes*; IL, interleukin; COX-2, cyclooxygenase-2; iNOS, inducible nitric oxide synthase; TNFα, tumor necrosis factor-α; SD, standard deviation.

To assess whether these metabolites attenuate *C. acnes*-induced inflammatory responses, KCs were stimulated with *C. acnes* (1 × 10^7^ CFU) and then treated with each metabolite. Quantitative reverse transcription–polymerase chain reaction (qRT-PCR) analysis revealed robust induction of COX2, iNOS, TNF-α, IL-1β, IL-6, and IL-8 in response to *C. acnes*, all of which were significantly suppressed by TAPI, IPA, and ILA (Figure 2). In contrast, IAA showed minimal or inconsistent inhibitory effects. Given that TAPI served as a positive control and that IAA demonstrated inferior anti-inflammatory activity compared with the other metabolites, IAA was excluded from in vivo experiments.

### 2.2. In Vivo Attenuation of C. acnes-Induced Skin Inflammation

**Figure 3:**
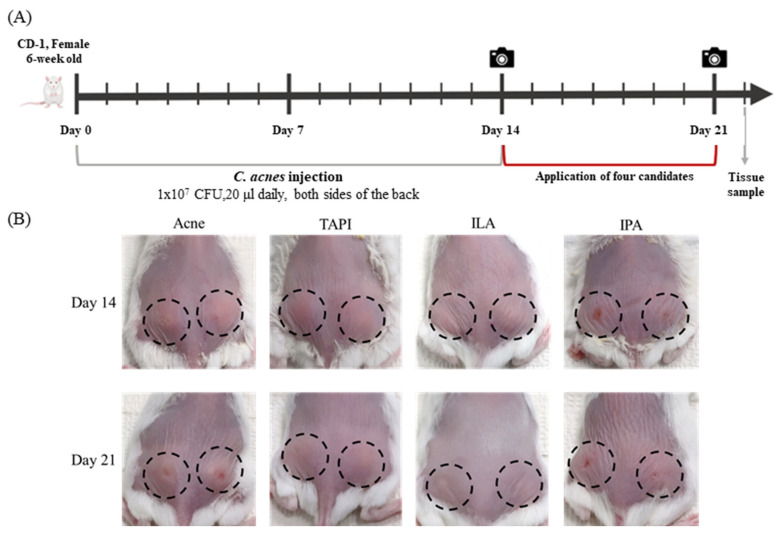
(**A**) A murine acne model was generated by intradermal injection of *C. acnes* (1 × 10^7^ CFU in 20 μL) into both sides of the dorsal skin for two weeks, followed by topical application of TAPI, IPA, or ILA for seven days. (**B**) Clinical assessment of mice dorsal skin after 14 and 21 days in the Acne group and the candidate metabolites groups. The camera symbols indicate the time of clinical assessment. The black dashed circles indicate the areas injected with *C. acnes* (1 × 10^7^ CFU in 20 μL). TAPI, tapinarof; IPA, indole-3-propionic acid; ILA, indole-3-lactic acid.

**Figure 4:**
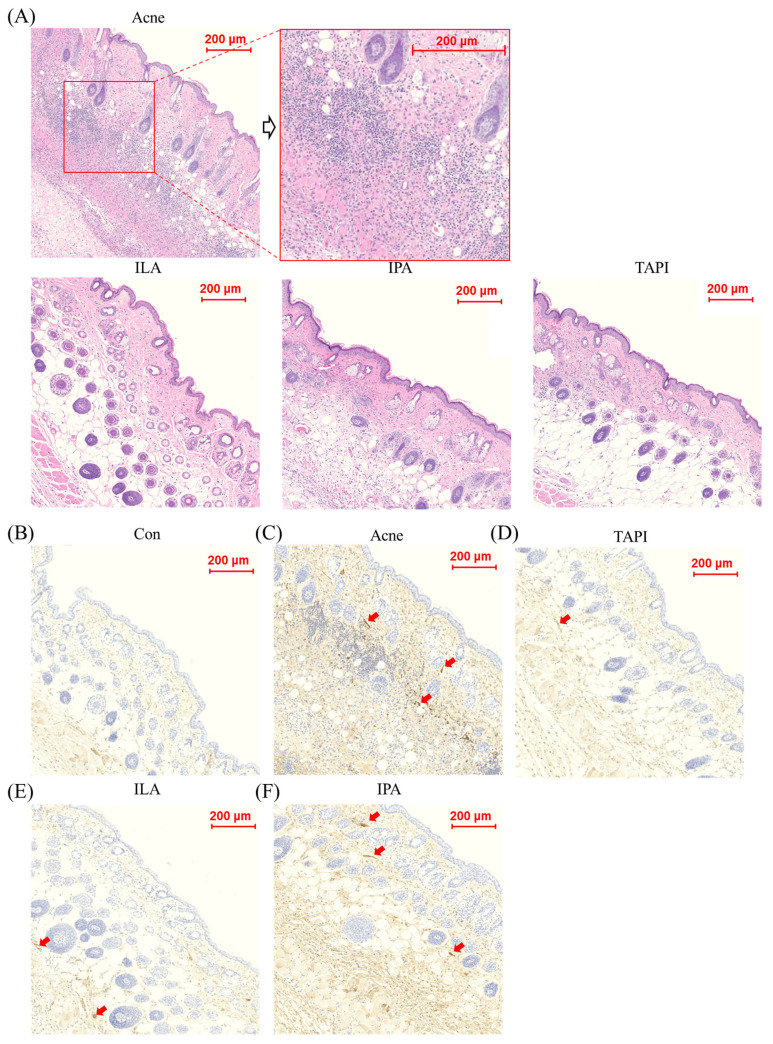
(**A**) Histological assessment after 21 days in the Acne group and the candidate metabolites groups, scale bar = 100 μm. (**B**–**F**) Immunohistochemical analysis of IL-1β expression in skin tissues from control, *C. acnes*-treated (Acne), and candidate-treated groups (TAPI, IPA, and ILA). Representative images show IL-1β staining in the dermal and perifollicular regions of (**B**) control, (**C**) *C. acnes*-treated, (**D**) TAPI-treated, (**E**) IPA-treated, and (**F**) ILA-treated skin. The Acne group exhibited markedly increased IL-1β staining intensity compared with the control group, whereas TAPI, used as a positive anti-inflammatory control, attenuated *C. acnes*-induced IL-1β expression. Notably, ILA showed the most pronounced suppression of IL-1β staining among the tested candidates. Arrows indicate representative areas of enhanced IL-1β expression in the dermis and perifollicular regions. Scale bar = 200 μm. CON, control; *C. acnes*, *Cutibacterium acnes*; IL-1β, interleukin-1 beta; TAPI, tapinarof; IPA, indole-3-propionic acid; ILA, indole-3-lactic acid.

A murine acne model was generated by intradermal injection of *C. acnes* (1 × 10^7^ CFU in 20 μL) into both sides of the dorsal skin for two weeks, followed by topical application of TAPI, IPA, or ILA for seven days (Figure 3A). By day 14, clinical assessment showed the appearance of a papule with persistent erythema and swelling at the injection site, which grew larger by day 21, whereas all treatment groups (except IPA) showed visible improvement (Figure 3B). Histological analysis revealed dense inflammatory-cell infiltration and marked dermal thickening in the Acne group, both of which were substantially reduced following treatment with the candidate metabolites (Figure 4A). Among them, ILA-treated lesions demonstrated the thinnest epidermis and the least inflammatory infiltration. Immunohistochemical analysis further supported these findings (Figure 4B–F). IL-1β expression was strongly detected in the epidermis and perifollicular dermis of the Acne group but was markedly diminished in all treatment groups, with the ILA group showing the greatest reduction. Collectively, these results indicate that the candidate metabolites—particularly ILA—mitigate *C. acnes*-induced inflammatory signaling in vivo.

### 2.3. Transcriptomic Remodeling by ILA in KC

**Figure 5:**
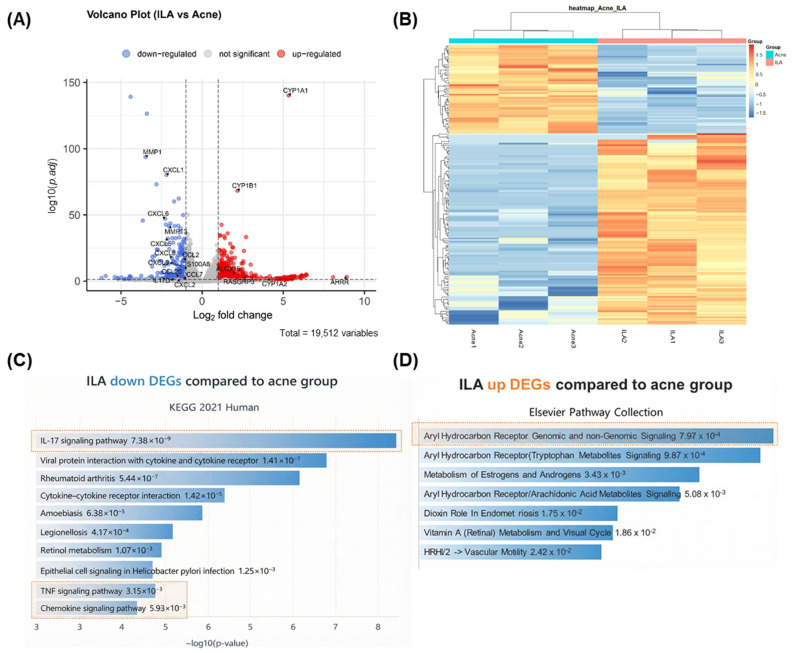
(**A**) Volcano plot showing DEGs between *C. acnes*-stimulated keratinocytes (Acne group) and ILA-treated cells. ILA markedly downregulated inflammatory genes (*CXCL1*, *CXCL2*, *CXCL8*, *MMP1*, *MMP3*, *PTGS2*) and upregulated AHR-related genes (*CYP1A1*, *CYP1B1*, *AHRR*). (**B**) Heatmap of DEGs demonstrating that ILA induces a transcriptional profile distinct from the Acne group, with broad suppression of inflammatory gene clusters. (**C**) Pathway enrichment analysis of downregulated DEGs showing inhibition of IL-17 signaling, cytokine–cytokine receptor interaction, TNF signaling, and chemokine signaling (indicated in orange boxes). (**D**) Upregulated DEGs were enriched in AHR-associated pathways, including tryptophan metabolite signaling (indicated in the orange box). ILA, indole-3-lactic acid; *C. acnes*, *Cutibacterium acnes*; DEG, differentially expressed gene; AHR, aryl hydrocarbon receptor.

Based on the in vitro and in vivo evidence demonstrating the anti-inflammatory activity of ILA, we next performed bulk RNA sequencing (bulk RNA-seq) to characterize its molecular effects in *C. acnes*-stimulated KCs. Bulk RNA-seq was conducted using KCs stimulated with *C. acnes* (Acne group) and subsequently treated with ILA. Volcano plot analysis revealed that multiple *C. acnes*-induced inflammatory genes—including *C-X-C Motif Chemokine Ligand* (*CXCL*) *1*, *CXCL2*, *CXCL8*, *Matrix Metalloproteinase* (*MMP*) *1*, *MMP3*, and *Prostaglandin-endoperoxide synthase 2* (*PTGS2*)—were markedly downregulated by ILA, whereas *Cytochrome* (*CYP*) *1A1*, *CYP1B1*, and *AHR Repressor* (*AHRR*) were significantly upregulated (Figure 5A). A heatmap of differentially expressed genes further confirmed that ILA-treated cells displayed a transcriptional profile distinct from the Acne group, characterized by broad suppression of inflammatory gene clusters (Figure 5B).

Pathway enrichment analysis of the downregulated differentially expressed genes (DEGs) demonstrated significant inhibition of inflammation- and immunity-related pathways, including IL-17 signaling, cytokine–cytokine receptor interaction, TNF signaling, and chemokine signaling (Figure 5C). In contrast, upregulated DEGs were enriched in AHR-associated pathways, such as those related to Trp metabolite signaling (Figure 5D). Collectively, these results indicate that ILA attenuates *C. acnes*-induced inflammatory responses in KCs while activating AHR-linked metabolic and homeostatic programs.

## 3. Discussion

In this study, we demonstrated that the microbiome-derived Trp metabolite ILA robustly attenuates *C. acnes*-induced inflammation in both KCs and a murine acne model. ILA markedly reduced the expression of key inflammatory cytokines and chemokines—including IL-1β, IL-6, IL-8, and TNF-α—upregulated by *C. acnes*, and it effectively decreased immune-cell infiltration and epidermal hyperplasia in vivo. Bulk RNA sequencing further showed that ILA suppresses major interferon- and IL-17–related inflammatory pathways while activating AHR-responsive transcriptional programs. Together, these findings highlight ILA as a potent anti-inflammatory metabolite that exerts its effects, at least in part, through AHR-mediated regulatory mechanisms, underscoring its potential as a therapeutic candidate for acne vulgaris.

Acne vulgaris is driven by a complex interplay of follicular hyperkeratinization, excess sebum production, microbial overgrowth, and innate immune activation. Among these factors, *C. acnes* plays a central role, particularly through its ability to activate Toll-like receptor (TLR) 2- and TLR4-mediated inflammatory pathways in KCs. This stimulation triggers downstream activation of NF-κB and Mitogen-activated protein kinase (MAPK) signaling, leading to increased production of IL-1β, IL-6, IL-8, TNF-α, COX2, and other chemokines from KCs, all of which contribute to the recruitment of neutrophils, dermal inflammation, and lesion formation [15,16]. IL-1β, in particular, promotes comedogenesis by driving Th17-mediated KC hyperproliferation [17], while IL-8 drives the intense neutrophilic infiltration characteristic of inflammatory papules and pustules [18]. In our study, these cytokines were markedly upregulated upon *C. acnes* stimulation, consistent with known acne pathogenesis. Treatment with the Trp metabolites reduced these inflammatory mediators and pathways, indicating a direct suppressive effect on the *C. acnes*-driven inflammatory cascade.

Mechanistically, our findings provide further evidence supporting the involvement of the Trp metabolite–AHR axis in cutaneous immune regulation. AHR is a ligand-activated transcription factor responsive to both endogenous metabolites and microbial indoles. Prior literature shows that AHR activation can suppress pro-inflammatory cytokines and promote barrier-stabilizing pathways in various chronic inflammatory skin conditions [11,12]. In acne, it has been suggested that Trp metabolites from the gut microbiota could ameliorate skin inflammation as well as modulate sebocyte activity [13]. In HS, impaired microbial Trp catabolism results in loss of bacterial indoles and defective AHR activation, leading to uncontrolled IL-1β-driven inflammation [14]. Our findings are consistent with these observations: the Trp-derived metabolite ILA suppressed *C. acnes*-induced inflammatory gene expression while activating AHR-dependent transcriptional programs in KCs. This suggests that the anti-inflammatory effects of ILA are mediated, at least in part, through AHR activation, supporting the notion that restoring microbial indole signaling can help rebalance cutaneous immunity. The concordance between our experimental results and prior AHR-centered dermatologic research further reinforces the mechanistic plausibility of microbial Trp metabolites as potential therapeutic agents.

The use of microbial products for acne treatment is emerging as a promising therapeutic direction. Approaches such as probiotics, postbiotics, bacteriophage therapy, and commensal transplantation have been explored [5]. Oral and topical probiotics containing *Lactobacillus* and *Bifidobacterium* strains have shown reductions in inflammatory lesions through modulation of systemic cytokines and improvements of the skin barrier [9,19]. Bacterial fermented lysates have alleviated acne severity, sebum production, and enhanced skin hydration in a previous clinical study [20]. Additionally, selective *C. acnes* bacteriophages are being developed to reduce pathogenic strains while preserving commensal diversity [21]. Compared with these strategies, our approach uniquely targets bacterial metabolic products rather than live microorganisms, offering advantages in stability, safety, and formulation. Trp metabolites provide a precise, mechanism-driven intervention that bypasses challenges associated with live microbiome therapies, such as colonization variability or failure. This positions ILA as a promising representative of a new class of microbiome-derived small molecules with substantial therapeutic potential.

This study has several limitations. First, although the in vivo mouse model is widely used, it does not fully recapitulate the hormonal influences or sebaceous gland physiology characteristic of human acne. Second, our experiments focused primarily on KCs, and the effects of ILA on other relevant cell types—such as sebocytes, fibroblasts, and immune cells—remain to be elucidated. Third, while RNA-seq revealed AHR-associated transcriptional changes, direct mechanistic studies (e.g., AHR knockdown or antagonist experiments) are necessary to definitively confirm AHR-dependent pathways. Lastly, dose–response relationships and pharmacokinetic profiles of each metabolite require further evaluation to support clinical translation. Future studies should incorporate AHR-specific mechanistic validation, sebocyte functional assays, human skin explant models, and ultimately early-phase clinical trials to determine safety, efficacy, and optimal delivery strategies.

## 4. Materials and Methods

### 4.1. C. acnes Culture

*C. acnes* strain ATCC 6919 (ATCC, Manassas, VA, USA) was cultivated in Reinforced Clostridial Medium (Oxoid, Hampshire, UK) under anaerobic conditions at 37 °C for approximately 72 h. After incubation, the cultures were centrifuged at 14,000 rpm for 20 min at 4 °C, and the pellets were rinsed twice with sterile phosphate-buffered saline (PBS). The bacterial suspension was then adjusted to an optical density of 1.0 at 600 nm, corresponding to 1 × 10^7^ CFU in a 50 μL volume. Fresh cultures were prepared weekly, stored at 4 °C, and used for daily applications throughout the experimental period.

### 4.2. Preparation of Candidate Metabolite

**Figure 6:**
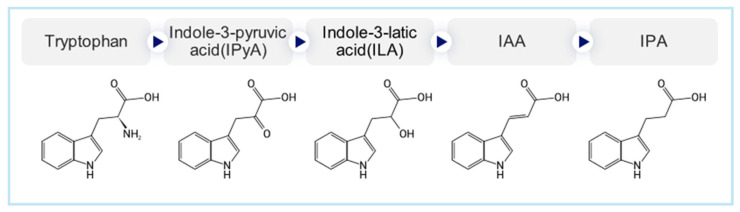
Structures of the selected small-molecule candidates, including indole-3-lactic acid (ILA), indole-3-acrylic acid (IAA), indole-3-propionic acid (IPA), and tapinarof.

ILA, IAA, and IPA were purchased from Millipore Sigma (St. Louis, MO, USA). (E)-3,5-Dihydroxy-4-isopropyl-trans-stilbene (Tapinarof) was provided by Prof. Hyun Bong Park (Gangneung-Wonju National University, Gangwon-do, Republic of Korea). All compounds, when needed, were prepared in DMSO. The structures of the compound are shown in Figure 6.

### 4.3. Cell Culture and Viability Assay

Primary human epidermal KCs (PCS-200-011, ATCC) were maintained in KBM Gold medium (Lonza, Basel, Switzerland) supplemented with the KGM Gold SingleQuot kit (Lonza). Cells were incubated at 37 °C in a humidified atmosphere of 5% CO_2_ and routinely subcultured with trypsin–EDTA at three-day intervals.

Cell viability of KC was analyzed using the CCK-8 reagent (Dojindo, Kumamoto, Japan). Cells were seeded into 96-well plates at 5 × 10^4^ cells per well and allowed to stabilize. After stabilization, *C. acnes* was added at a final concentration of 1 × 10^7^ CFU to induce acne-like inflammatory activation. For testing, cultures were treated with increasing concentrations of each metabolite: ILA (0.25, 0.5, 1, 2, and 4 mM), IAA (0.25, 0.5, 1, 2, and 4 mM), IPA (0.25, 0.5, 1, 2, and 4 mM), and TAPI (1.25, 2.5, 5, 10, and 20 µM). After incubation, absorbance at 450 nm was read on a VersaMax microplate reader (Molecular Devices, San Jose, CA, USA). Cell viability was calculated as a percentage relative to the untreated control, and all experiments were performed at least three times.

### 4.4. In Vivo C. acnes-Induced Acne Model and Treatment

Female CD-1 mice aged six weeks (OrientBio, Seongnam, Republic of Korea) were housed under controlled environmental conditions (24 ± 0.5 °C, 55–65% humidity, 12-h light/dark cycle) with unrestricted access to food and water. After a one-week adaptation period, the animals were randomly divided into five experimental groups (n = 5 for each). In total, 25 mice were included in the study, comprising one control group and four experimental groups receiving individual metabolites following *C. acnes* induction.

To induce acne-like inflammation, *C. acnes* (1 × 10^7^ CFU in 20 µL) was injected intradermally into both sides of the shaved dorsal skin once daily for 14 days as previously described [22]. Beginning on day 15, each metabolite was topically applied to the dorsal skin once daily for seven consecutive days. The treatment solutions were prepared at the following final concentrations: ILA, 4 mM; IPA, 4 mM; IAA, 2 mM; and TAPI, 20 µM. All compounds were dissolved in a mixed solvent composed of 10% DMSO, 40% PEG 300, 5% Tween-80, and 45% saline to ensure complete solubilization and consistent application. Immediately after application, the treated area was gently tapped approximately 40 times with a sterile plastic spatula to facilitate uniform topical absorption, while mice were lightly anesthetized with inhalational isoflurane (100 mL; Isoflurane, Hana Pharm Co., Ltd., Seoul, Republic of Korea), as previously described [22]. Macroscopic skin changes were documented on days 14 and 21. On day 22, animals were euthanized by CO_2_ inhalation in accordance with the ARRIVE guidelines and the institutional animal care protocol (IACUC approval no. 2023-0111), and dorsal back skin tissues were harvested for subsequent histological analyses.

### 4.5. Histopathology and Immunohistochemistry

Dorsal skin specimens collected immediately after euthanasia were fixed in 10% neutral-buffered formalin, dehydrated through graded ethanol, cleared in xylene, and embedded in paraffin. 4-µm sections were prepared using a rotary microtome. For histological evaluation, hematoxylin-stained sections were examined to assess epidermal and dermal architecture.

For immunohistochemical analysis, paraffin-embedded sections underwent heat-mediated antigen retrieval in EnVision FLEX TRS High pH buffer (S2367, Dako, Glostrup, Denmark) for 30 min, followed by quenching of endogenous peroxidase activity with 3% hydrogen peroxide on ice. After blocking nonspecific binding with 5% bovine serum albumin, slides were incubated overnight at 4 °C with a primary antibody against IL-1β (1:500, Cell Signaling Technology, Danvers, MA, USA). Antibody binding was visualized using the DAKO peroxidase/DAB detection system, and nuclei were counterstained with Mayer’s hematoxylin. All staining steps were performed in parallel under identical conditions for each experimental group.

### 4.6. Quantitative Reverse Transcription–Polymerase Chain Reaction (qRT-PCR)

To determine the anti-inflammatory effects of each metabolite, KCs were first exposed to *C. acnes* (1 × 10^7^ CFU) for 24 h as previously reported [22]. After stimulation, the culture medium was replaced with fresh medium containing the respective metabolite, and cells were further incubated for an additional 24 h. Total RNA was extracted using RNAiso Plus reagent (Takara Bio, Shiga, Japan) according to the manufacturer’s protocol. The concentration and purity of RNA were verified spectrophotometrically, and complementary DNA (cDNA) was synthesized using the RNA to cDNA EcoDry™ premix kit (Takara Bio). Quantitative PCR was performed with SYBR Green Master Mix (Promega, Madison, WI, USA) on a QuantStudio 3 Real-Time PCR System (Applied Biosystems, Foster City, CA, USA). Relative mRNA levels were normalized to GAPDH and analyzed by the 2^−ΔΔCt^ method. The primer sequences used in this study are listed in Table 1.

### 4.7. Bulk RNA-Seq

For transcriptomic profiling, total RNA was extracted from KCs treated under the same sequential protocol described above (*C. acnes* exposure for 24 h → medium replacement → metabolite treatment for 24 h). RNA integrity was confirmed before library preparation using a TruSeq RNA Library Prep Kit (Illumina, San Diego, CA, USA). Sequencing was conducted in paired-end 100 bp mode on the NextSeq 550 platform (Macrogen, Seoul, Republic of Korea). Low-quality reads were trimmed and aligned to the human reference genome with HISAT2. Gene-level abundance estimates (read counts, FPKM, and TPM) were obtained using StringTie, and differential expression was evaluated with DESeq2 applying thresholds of |fold change| ≥ 2 and *p* < 0.05.

### 4.8. Statistical Analysis

We expressed all experimental data as the mean ± standard deviation (SD). Statistical analyses were conducted using SPSS software (version 25.0; IBM Corp., Armonk, NY, USA). We first verified the normality and homogeneity of variance prior to statistical testing. For pairwise comparisons, we applied unpaired Student’s *t*-tests when these assumptions were satisfied. Differences between each treatment group and the *C. acnes*-only control were analyzed using independent *t*-tests. We analyzed differences among multiple experimental groups using one-way analysis of variance, followed by Tukey’s honestly significant difference post hoc test for multiple comparisons. A *p* value < 0.05 was considered statistically significant. We also applied the Benjamini–Hochberg procedure to control the False Discovery Rate (FDR) in our DESeq2 analysis and used adjusted *p*-values for all transcriptomic comparisons.

## 5. Conclusions

This study demonstrates that the microbiome-derived Trp metabolite ILA effectively suppresses *C. acnes*-induced inflammation by reducing key inflammatory mediators, attenuating immune-cell infiltration and epidermal hyperplasia, and transcriptionally inhibiting cytokine-driven inflammatory pathways while activating AHR-associated homeostatic programs. Together, these findings identify ILA as a promising postbiotic small molecule that modulates the Trp-derivative–AHR axis to restore cutaneous immune balance in acne vulgaris.

## Figures and Tables

**Table 1:** Sequence of primers for qRT-PCR for mouse and human genes.

Genes	Sequence
*TNFα*	Forward: 5′-CTCTTCTGCCTGCTGCACTTTG-3′ Reverse: 5′-ATGGGCTACAGGCTTGTCACTC-3′
*IL1β*	Forward: 5′-CCACAGACCTTCCAGGAGAATG-3′ Reverse: 5′-GTGCAGTTCAGTGATCGTACAGG-3′
*IL6*	Forward: 5′-AGACAGCCACTCACCTCTTCAG-3′ Reverse: 5′-TTCTGCCAGTGCCTCTTTGCTG-3′
*IL8*	Forward: 5′-GACCACACTGCGCCAACAC-3′ Reverse: 5′-CTTCTCCACAACCCTCTGCAC-3′
*COX2*	Forward: 5′-CGGTGAAACTCTGGCTAGACAG-3′ Reverse: 5′-GCAAACCGTAGATGCTCAGGGA-3′
*iNOS*	Forward: 5′-GCTCTACACCTCCAATGTGACC-3′ Reverse: 5′-CTGCCGAGATTTGAGCCTCATG-3′
*GAPDH*	Forward: 5′-TGTTGCCATCAATGACCCCTT-3′ Reverse: 5′-CTCCACGACGTACTCAGCG-3′

qRT-PCR, quantitative reverse transcription–polymerase chain reaction; TNFα, tumor necrosis factor-alpha; IL, interleukin; COX2, cyclooxygenase2; iNOS, inducible nitric oxide synthase.

## Data Availability

The raw data supporting the conclusions of this article will be made available by the authors on request. The datasets analyzed during the current study are available in the Gene Expression Omnibus repository. URL: https://www.ncbi.nlm.nih.gov/geo/query/acc.cgi?acc=GSE315350 (accessed on 5 January 2026).

## Abbreviations

The following abbreviations are used in this manuscript:

AHR	Aryl hydrocarbon receptor
*C. acnes*	*Cutibacterium acnes*
COX2	Cyclooxygenase 2
CXCL	Chemokine (C-X-C motif) ligand
IAA	Indole-3-acrylic acid
IL	Interleukin
ILA	Indole-3-lactic acid
iNOS	Inducible Nitric Oxide Synthase
IPA	Indole-3-propionic acid
KC	Keratinocyte
MAPK	Mitogen-activated protein kinase
MMP	Metalloproteinase
PTGS2	Prostaglandin-endoperoxide synthase 2
RNA-seq	RNA sequencing
TAPI	Tapinarof
TLR	Toll-like receptor
TNF-α	Tumor Necrosis Factor-alpha
Trp	Tryptophan
